# The use of intrathecal morphine in non-abdominal surgery: a scoping review

**DOI:** 10.1016/j.bjao.2025.100387

**Published:** 2025-03-20

**Authors:** Aart Jan W. Teunissen, Lieke van Gastel, Robert J. Stolker, Seppe A. Koopman

**Affiliations:** 1 Maasstad Hospital, Anaesthesiology, Rotterdam, the Netherlands; 2 Erasmus Medical Centre, University Medical Centre Rotterdam, the Netherlands

**Keywords:** intrathecal morphine, orthopaedic surgery, postoperative nausea and vomiting, pulmonary complications, pruritus, spine surgery, thoracic surgery, urinary retention

## Abstract

**Background:**

Intrathecal morphine can reduce pain and opioid requirements needed for postoperative pain relief. It can potentially aid in the effectiveness of enhanced recovery protocols in non-abdominal surgery. However, concerns about side-effects may have hindered its use. This scoping review evaluates the effectiveness, appropriate dosage, and adverse effects of intrathecal morphine in non-abdominal surgery.

**Methods:**

We systematically searched for randomised controlled trials examining the use of intrathecal morphine in non-abdominal surgery.

**Results:**

The search identified 75 trials involving 4685 patients. We undertook a scoping review of these randomised controlled trials, including bias assessments, to comprehensively analyse the effectiveness and side-effects of intrathecal morphine. The findings indicate that intrathecal morphine reduced postoperative pain and opioid consumption after spinal surgery, thoracic surgery, and orthopaedic lower extremity surgery. However, it was associated with an increased incidence of itching, postoperative nausea and vomiting, and urinary retention, particularly in orthopaedic procedures. Delayed respiratory depression was absent with low to moderate doses (<500 μg) in the reviewed studies.

**Conclusions:**

This review supports the effectiveness of intrathecal morphine in non-abdominal surgery. However, the benefits must be carefully weighed against potential side-effects that could lead to prolonged hospital stays.

**Clinical trial registration:**

PROSPERO-registry CRD42021233936.

Despite the known importance, inadequate postoperative pain treatment remains a problem, with an incidence of up to 80%.^1^ Adequate postoperative analgesia is vital for adequate postoperative recovery and patient satisfaction. Therefore, adequate analgesia is crucial in widely implemented enhanced recovery programmes.^2^ Despite growing concerns about side-effects and addiction, opioids remain important in postoperative pain management.^3^ Side-effects include respiratory depression, urinary retention, constipation, nausea and vomiting.^4^

A technique that has received renewed interest is (single-shot) intrathecal morphine administration, either alone or added to conventional intrathecal anaesthesia. The first case series was reported in 1979.^5^ It is a relatively straightforward procedure with low technical failure rates.^6^ As morphine is a relatively hydrophilic opioid, intrathecal administration provides long-lasting pain relief, relieving postoperative pain for up to 48 h.^7^

A recently published meta-analysis in laparoscopic and open abdominal surgery confirmed improved postoperative pain control, lower systemic morphine consumption, and shorter stay with intrathecal morphine.^8^

Uncertainty about the incidence and concern about adverse effects may have caused a decline in intrathecal morphine usage.^9^ Known adverse effects are postoperative nausea and vomiting (PONV), urinary retention, and pruritus.^4^^,^^10^^,^^11^ These side-effects seem dose-dependent and can be prevented or treated effectively.^7^^,^^10^, ^11^, ^12^, ^13^ A rare but serious adverse effect is (late) respiratory depression. A recent meta-analysis reported late respiratory depression to occur only when using high doses of morphine (>500 μg).^8^ This meta-analysis, however, only studied abdominal surgery. With this scoping review, we intend to verify if this also applies to non-abdominal surgery.

To date, several reviews and meta-analyses reported on the use of intrathecal morphine in non-abdominal surgery.^14^, ^15^, ^16^, ^17^, ^18^, ^19^, ^20^ These studies focused mainly on one type of surgery, comparing only one anaesthesia technique or placebo with intrathecal morphine. Furthermore, they reported on outcomes other than pain scores, which may limit the generalisability of the efficacy of intrathecal morphine to different types of surgery. Performing a review on intrathecal morphine use in different types of surgery provides a broader understanding of the efficacy of intrathecal morphine in non-abdominal surgery. Furthermore, several studies were published after publication of the aforementioned meta-analyses.

This scoping review aimed to evaluate the efficacy and safety of intrathecal morphine in reducing postoperative pain intensity or postoperative opioid use in patients undergoing any type of surgery, exempting abdominal surgery.

## Methods

This study followed the PRISMA for Scoping Reviews (PRISMA-ScR) statement for reporting scoping reviews.^21^ The protocol was registered prospectively in the PROSPERO database, registration number CRD42021233936.^22^

### Literature search

Literature searches were conducted using PubMed, Embase, Cochrane, Web of Science, and Google Scholar. For PubMed, the MeSH-terms intraspinal injection, intrathecal injection, spinal anaesthesia, morphine, postoperative pain, pain measurement, general surgery, surgical procedure, operative or surgery were used. For Embase, an all-fields search was performed using the terms intrathecal drug administration, morphine, and pain score. A title search was conducted for Google Scholar, using the terms intrathecal morphine and pain, screening all articles published between 1999 and 2024. We did look into older randomised controlled trials (RCTs) through the cited systematic reviews to understand what impact high-dose intrathecal morphine had. However, this is no longer a contemporary practice, so we decided to limit our review to practice over the past 25 yr. A free text search was performed for all databases, using the terms intrathecal morphine and pain scores. Meta-analyses and (systematic) reviews were scanned for additional studies not found in our literature search.^7^^,^^10^^,^^14^, ^15^, ^16^, ^17^, ^18^^,^^20^^,^^23^, ^24^, ^25^

### Inclusion and exclusion criteria

RCTs comparing the efficacy of intrathecal morphine with other analgesic regimens or placebo in adult humans undergoing spinal, cardiac, thoracic, vascular or orthopaedic surgery were eligible for inclusion. Efficacy was measured using pain scores or a reduction of opioid use. Trials in a non-surgical population (i.e. chronic pain), abdominal surgery, and trials comparing intrathecal morphine with other intrathecal administered opioids were excluded. An article was also excluded if the full text was unavailable in English or Dutch.

### Data extraction

Two authors (LG and AJT) independently screened the title and abstract, extracted data into an Excel file (Microsoft Excel, Redmond, WA, USA) and assessed all eligible articles for risk of bias. Any discrepancies were independently resolved by the last author (SK).

For the included articles, the first author, year of publication, type of study (RCT, systematic review or meta-analysis) and the type of surgery were recorded. The study groups, number of patients, the anaesthetic technique in the control group, the type of intrathecal opioid in the intervention group, and the dose of intrathecal morphine used were registered. If used, co-medication was noted. The type of pain score used (visual analogue scale [VAS], numerical rating scale, and other types of rating scales) and the duration of follow-up were collected. Pain scores were converted to a 1 to 10 scale for easier comparison. The duration of action was extracted from the articles. Most articles defined the duration of action from the moment of administration until the moment the pain scores started increasing. If provided, secondary outcomes such as adverse events, supplementary opioid requirement, or length of hospital stay were recorded. All included articles were checked for possible conflicts of interest and were evaluated using the Cochrane Risk of Bias Tools for Randomized Controlled Trials version 2.^26^^,^^27^

### Outcome measures

In this scoping review, a reduction of postoperative pain and postoperative opioid use was considered as the efficacy of intrathecal morphine. Efficacy was defined as a difference in postoperative pain or opioid consumption between patients receiving intrathecal morphine and the control group.^25^ Adverse effects of intrathecal morphine were extracted, such as respiratory depression, nausea, vomiting, urinary retention, and pruritus. Side-effects were dichotomously reported in most of the included studies.

## Results

**Fig 1:**
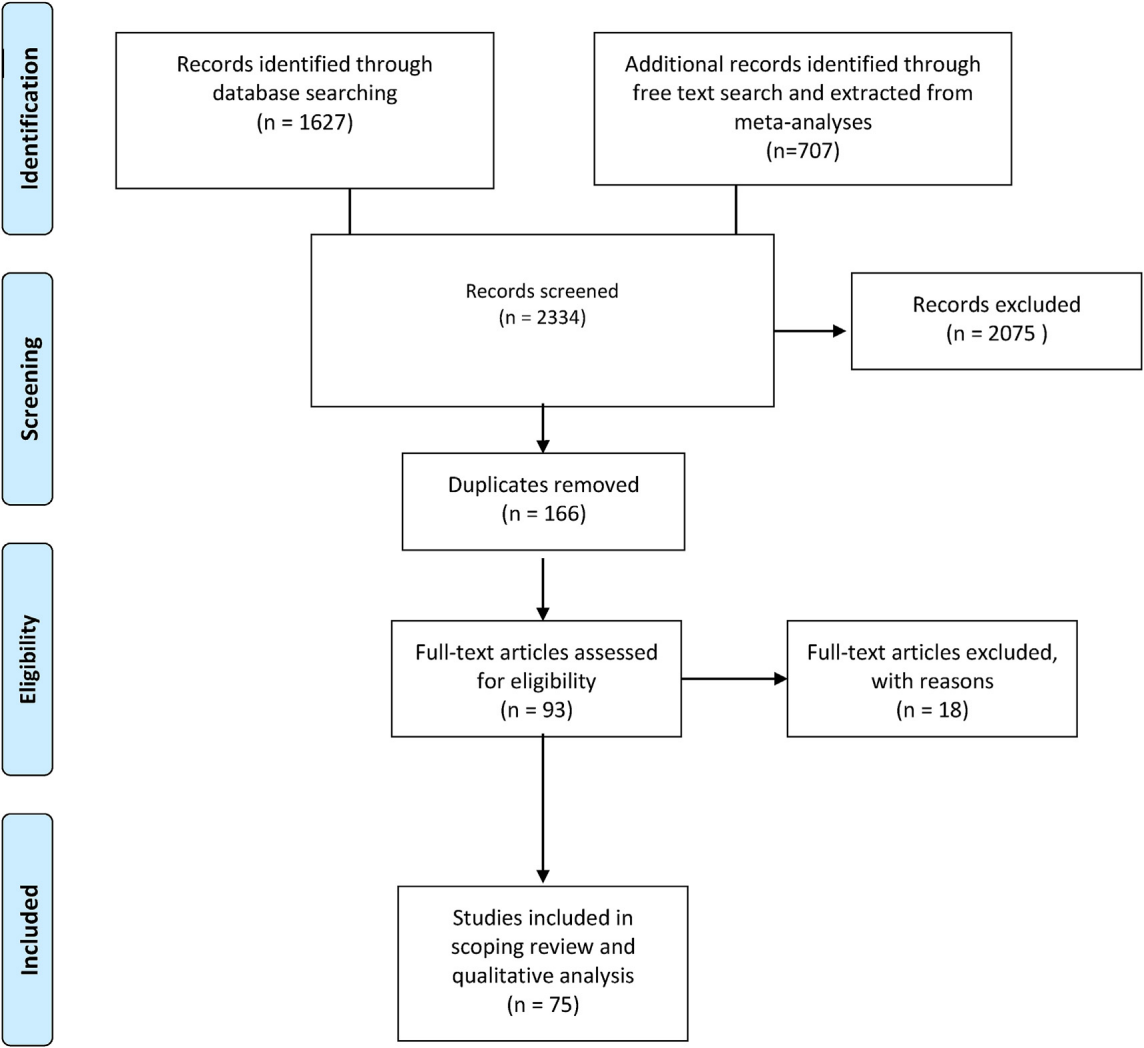
Flowchart presenting the scoping review process.

From all databases, 2137 articles were screened for eligibility. A flowchart of the in- and exclusion process is shown in Fig 1. After full-text screening and excluding all duplicates, 75 articles with 4685 patients were included in this scoping review. The included articles were then divided into three groups to provide a structured overview: orthopaedic surgery of the lower extremity, spinal surgery, and thoracic surgery. The data extraction table with detailed study characteristics can be found in Supplementary Tables S1–S3.

The methodological assessment was performed, as shown in Supplementary Table S1. The overall risk of bias was intermediate to high because of the lack of blinding of participants, selection bias arising from absent registration in appropriate databases, relatively small population sizes, or insufficient details regarding the randomisation process, as shown in Supplementary Table S2. The frequency of measurement of pain scores and the registration of side-effects differed between the studies.

### Spine surgery

Ten studies investigated intrathecal morphine in spinal surgery, with 715 patients in total. The studies were divided according to the type of spinal surgery (Table 1). The control group received general anaesthesia alone or general anaesthesia combined with epidural anaesthesia.

**Table 1:** The efficacy comparing intrathecal morphine with control in different types of spinal surgery: laminectomy and discectomy, and posterior spinal fusion.

Type of surgery	Number of studies (*n*)	Number of patients (*n*)	Control group treatment in studies	Median dose of morphine (mg)	Median duration of effect (h)	Pain score	Opioid use
Laminectomy and discectomy	N = 5	N = 358	GA (*n* = 2) EA/CSE (*n* = 1) GA+EA/PA (*n* = 1) SA ropivacaine 7.5 mg (*n* = 1)	0.2 mg (0.1 mg–3.5 mcg/kg)	24 (8–24)	Higher: 1 Similar: 1 Lower: 3	Higher: 1 Similar: 1 Lower: 3
Posterior spinal fusion	N = 5	N = 357	GA (*n* = 5)	0.25 mg (0.1 mg–15mcg/kg)	24 (8–48)	Lower:5	Lower:5

CSE, combined spinal epidural; EA, epidural anaesthesia; GA, general anaesthesia; PA, paraspinal anaesthesia; SA, spinal anaesthesia.

Studies showed varying results for laminectomy and discectomy (*n*=5) (Table 1).^28^, ^29^, ^30^, ^31^, ^32^ Four studies showed higher efficacy of intrathecal morphine compared with general anaesthesia or intrathecal local anaesthetic agents.^28^^,^^29^^,^^31^^,^^32^ There was a pain score reduction of 1–2 points.^28^^,^^29^^,^^31^^,^^32^ The reported reduction in the need for rescue pain treatment, including opioids, varied from 0% to 66%.^28^^,^^29^^,^^31^^,^^32^ The study reporting a lower efficacy showed a higher pain score of 1 point compared with epidural anaesthesia with 50% higher opioid use.^30^

For posterior spinal fusion, all five studies reported a higher efficacy of intrathecal morphine compared with the control group.^33^, ^34^, ^35^, ^36^, ^37^ They showed a reduction in postoperative pain scores ranging from 1 to 2.5 and a decrease in opioid use ranging from 17% to 50%.^33^, ^34^, ^35^, ^36^, ^37^

Eight trials found no significant differences in the incidence of adverse events between groups.^28^^,^^31^, ^32^, ^33^^,^^35^, ^36^, ^37^ One study reported a higher incidence of pruritus and PONV in the intrathecal morphine group.^30^ One study reported only a higher incidence of pruritus.^29^ Eight studies reported the incidence of respiratory depression, reporting either no cases or a lower incidence in the intrathecal morphine group (seven studies).^28^^,^^30^, ^31^, ^32^, ^33^^,^^35^^,^^37^ One study reported one patient with early onset respiratory depression presenting with hypoxaemia (<90%) and bradypnoea (<10 bpm) at 1 h after operation without any other symptoms for >30 s, for which he received naloxone.^29^

#### Thoracic surgery

Twenty-five studies investigated intrathecal morphine in thoracic surgery with a total of 1152 patients.^38^, ^39^, ^40^, ^41^, ^42^, ^43^, ^44^, ^45^, ^46^, ^47^, ^48^, ^49^, ^50^, ^51^, ^52^, ^53^, ^54^, ^55^, ^56^, ^57^, ^58^, ^59^, ^60^, ^61^ Subdivisions according to type of surgery were made for sternotomy, thoracotomy, and minimally invasive cardiac or thoracoscopic surgery (Table 2).

**Table 2:** The efficacy and incidence of adverse events comparing intrathecal morphine with control in different types of thoracic surgery: cardiac surgery, thoracotomy, and minimally invasive cardiac surgery.

Type of surgery	Number of studies (*n*)	Number of patients (*n*)	Control group treatment^a^	Median dose of intrathecal morphine (mg)	Median duration of effect (h)	Pain score	Opioid use
Sternotomy	15	671	GA (*n*=15)	0.5 mg (1.5 μg kg^−1^–2 mg)	24 (2–48)	Similar: 2 Lower: 13	Similar: 1 Lower: 14
Thoracotomy	6	281	GA (*n*=3) EA (*n*=2) cPVB (*n*=21) IT sufentanil (*n*=1)	0.5 mg (0.2–1 mg)	36 (0–48)	Higher: 1 Similar: 2 Lower: 3	Higher: 0 Similar: 2 Lower: 4
Minimally invasive cardiac or thoracoscopic surgery	3	200	GA (*n*=3)	0.4 mg 3–5 μg kg^−1^	36 (1–72)	Lower: 3	Lower: 3

cPVB, continuous paravertebral block; EA, epidural anaesthesia; GA, general anaesthesia; IT, intrathecal.

Cardiac surgery includes coronary artery bypass grafting (CABG), open valve replacement, minimally invasive cardiac surgery.

a All neuraxial and locoregional techniques were combined with general anaesthesia.

For sternotomy (*n*=15), all studies showed higher efficacy of intrathecal morphine with general anaesthesia compared with general anaesthesia without intrathecal morphine as the control group.^40^^,^^42^, ^43^, ^44^^,^^46^, ^47^, ^48^, ^49^^,^^51^^,^^52^^,^^56^, ^57^, ^58^, ^59^, ^60^ Thirteen studies found lower pain scores. Fourteen found lower opioid use compared with control. The pain scores were 1–3 points lower.^44^^,^^46^, ^47^, ^48^, ^49^^,^^51^^,^^52^^,^^56^, ^57^, ^58^, ^59^, ^60^^,^^62^^,^^63^ Opioid consumption was 35–86% lower.^42^, ^43^, ^44^^,^^46^, ^47^, ^48^, ^49^^,^^51^^,^^52^^,^^57^, ^58^, ^59^, ^60^^,^^62^^,^^63^

For thoracotomy (six studies in total), three studies found superior efficacy of intrathecal morphine combined with general anaesthesia compared with general anaesthesia without intrathecal morphine as the control group. The pain scores were 2–3 points lower, and the opioid use 50–90% lower compared with general anaesthesia alone.^38^^,^^53^^,^^54^ One study comparing high-dose intrathecal morphine adjusted to patients' need and general anaesthesia with thoracic epidural anaesthesia and general anaesthesia showed higher efficacy with a lower opioid use of 87% in the intrathecal morphine group.^55^ A study comparing a group with a combination of intrathecal morphine, a continuous paravertebral block, and general anaesthesia with a group receiving a paravertebral block and general anaesthesia showed similar efficacy.^61^ Another study comparing a combination of intrathecal morphine, continuous paravertebral block, and general anaesthesia with a combination of thoracic epidural anaesthesia and general anaesthesia showed a lower efficacy of the intrathecal morphine group with a 1 point increased VAS score.^45^

All minimally invasive surgery studies showed higher efficacy of intrathecal morphine combined with general anaesthesia compared with general anaesthesia alone. The reduction in pain scores was 2–5 points and the reduction in opioid use was 50–100%.^39^^,^^41^^,^^50^ Twenty studies reported a similar incidence of adverse events (pruritus or PONV) compared with control.^38^, ^39^, ^40^^,^^42^, ^43^, ^44^, ^45^^,^^51^, ^52^, ^53^, ^54^, ^55^, ^56^, ^57^, ^58^, ^59^, ^60^, ^61^ Only one study reported an incidence of early respiratory depression with intrathecal morphine 8 μg kg^−1^.^56^ The other studies found no cases of respiratory depression (*n*=11) or a similar incidence between groups (*n*=9). One study reported a case of respiratory depression in the intrathecal morphine group because a dose of piritramide was given by mistake.^39^

### Orthopaedic surgery

Forty trials on intrathecal morphine in orthopaedic surgery of the lower extremity, with 2818 patients in total were included.^12^^,^^13^^,^^64^, ^65^, ^66^, ^67^, ^68^, ^69^, ^70^, ^71^, ^72^, ^73^, ^74^, ^75^, ^76^, ^77^, ^78^, ^79^, ^80^, ^81^, ^82^, ^83^, ^84^, ^85^, ^86^, ^87^, ^88^, ^89^, ^90^, ^91^, ^92^, ^93^, ^94^, ^95^, ^96^, ^97^, ^98^, ^99^, ^100^, ^101^ A subdivision of types of orthopaedic surgery was made (Table 3).

**Table 3:** The efficacy and incidence of adverse events comparing intrathecal morphine with control in different types of orthopaedic surgery: total knee arthroplasty (TKA), total hip arthroplasty (THA), lower extremity joint arthroplasty (combined population of patients undergoing TKA or THA), arthroscopic knee surgery, and trauma surgery of the lower extremity.

Type of surgery	Number of studies (*n*)	Number of patients (n)	Control group treatment in studies^a^	Median dose of morphine (mg)	Median duration of effect (h)	Pain score	Opioid use
Total knee arthroplasty	20	1422	SA (*n*=6) (c)LIA (*n*=3) PMDI (*n*=2) ACB (*n*=1) (c)FNB (*n*=6) c3-in-1 block (*n*=1) GA+LIA (*n*=1)	0.2 mg (0.035–0.3 mg)	24 (4–48)	Higher: 4 Similar: 3 Lower: 13	Higher: 2 Similar: 6 Lower: 12
Total hip arthroplasty	10	686	SA (*n*=2) SA low morph (*n*=1) FIB (*n*=1) cLIA (*n*=2) SA+GA + cLPB (*n*=1) GA+PCB (*n*=3)	0.1 mg (0.05–0.2 mg)	10 (6–18)	Higher: 2 Similar: 4 Lower: 4	Higher: 0 Similar: 3 Lower: 7
Total hip or knee arthroplasty	4	355	SA (*n*=4)	0.175 mg (0.05–0.2 mg)	24 (8–24)	Lower: 4	Lower: 4
Arthroscopic knee surgery	3	180	SA (*n*=2) SA+IAM (*n*=1)	0.175 mg (0.05–0.3 mg)	12 (6–12)	Similar: 1 Lower: 2	Higher: 1 Similar: 1 Lower: 1
Trauma surgery on the lower extremity	3	175	SA (*n*=2) SA+FNB (*n*=1)	0.2 mg (0.1 mg–5 μg kg^−1^)	36 (12–48)	Similar:1 Lower: 2	Lower: 3

ACB, adductor canal block; c3-in-1 block, continuous 3-in-1 block; (c)FNB, (continuous) femoral nerve block; (c)LIA, (continuous) local infiltration analgesia; cLPB, continuous lumbar plexus block; EA, epidural anaesthesia; FIB, fascia iliaca block; GA, general anaesthesia; IAM, intraarticular morphine; PAI, periarticular injection; PCB, psoas compartment block; PMDI, periarticular multimodal drug injection; PNB, posterior nerve block; SA, spinal anaesthesia (not combined with another anaesthetic technique).

a All peripheral nerve blocks were combined with spinal anaesthesia unless mentioned otherwise.

Twenty-nine trials reported a higher efficacy in the intrathecal morphine group.^7^^,^^13^^,^^66^^,^^68^, ^69^, ^70^^,^^72^^,^^75^^,^^77^, ^78^, ^79^^,^^81^, ^82^, ^83^, ^84^^,^^86^^,^^87^^,^^89^, ^90^, ^91^, ^92^, ^93^, ^94^, ^95^^,^^97^, ^98^, ^99^^,^^101^^,^^102^

Thirteen studies of total knee arthroplasty (TKA) found lower pain scores with intrathecal morphine compared with intrathecal local anaesthetics only.^67^, ^68^, ^69^^,^^76^^,^^77^^,^^79^^,^^81^^,^^86^^,^^89^^,^^92^^,^^95^^,^^97^, ^101^ The pain score reduction was 1–4 points, with the largest difference where no other regional technique with local anaesthetic was added. Twelve studies also found lower postoperative opioid use in the intrathecal morphine group with a reduction of 23–80%.^68^^,^^69^^,^^76^^,^^77^^,^^79^^,^^81^^,^^86^^,^^89^^,^^92^^,^^95^^,^^97^, ^101^ Again, the largest difference was reported if no other regional technique was added. In two studies comparing intrathecal morphine with a continuous femoral nerve block, the pain scores in the intrathecal morphine group were either higher (1.5 points) or similar but with higher use of opioids (70%).^66^^,^^88^ The VAS scores were higher (2 points) with increased opioid use (up to 50%) in the intrathecal morphine group compared with local infiltration analgesia (two studies).^71^^,^^85^

For total hip arthroplasty (10 studies), four studies reported a higher efficacy of intrathecal morphine compared with intrathecal local anaesthetics or general anaesthesia. The pain score reduction was 2–5 points, with a decrease in opioid use of 50–94%. A fascia iliaca plane block did not make a difference.^13^^,^^78^^,^^94^^,^^99^ A similar efficacy was found in four trials.^12^^,^^72^^,^^73^^,^^91^

A lower efficacy of intrathecal morphine with spinal anaesthesia was found when compared with local infiltration anaesthesia and spinal anaesthesia, with higher pain scores after 8 h and no reduction of opioid use.^65^^,^^80^

Higher efficacy of intrathecal morphine with spinal anaesthesia when compared with only spinal anaesthesia was found in all studies for total hip or knee joint arthroplasty combined (lower joint arthroplasty) with pain scores 1–3 points lower and opioid reduction of 33–90%.^72^^,^^87^^,^^90^^,^^100^

In arthroscopic knee surgery, pain scores were lower in all studies compared with control.^64^^,^^70^^,^^75^ Opioid use was only lower in one study (62%).^70^ When compared with intra-articular morphine, opioid consumption was 50% higher in the intrathecal morphine group.^64^

In other types of lower extremity surgery (trauma surgery), higher efficacy was reported in three trials.^84^^,^^98^^,^^102^ Intrathecal morphine yielded a pain reduction of 0.5 to 2 points in two studies^84^^,^^98^ and a similar pain score in one study.^102^ It gave a decrease in opioid use of 40–50%.^84^^,^^98^

Twenty-two studies showed a higher incidence of adverse events in the intrathecal morphine group, particularly pruritus and urinary retention.^12^^,^^13^^,^^64^^,^^66^^,^^67^^,^^72^, ^73^, ^74^, ^75^^,^^80^^,^^87^, ^88^, ^89^, ^90^^,^^92^^,^^94^^,^^96^, ^97^, ^98^^,^^100^, ^101^ Twenty-four trials reported no cases of respiratory depression.^12^^,^^13^^,^^64^^,^^66^^,^^67^^,^^70^^,^^71^^,^^73^^,^^74^^,^^78^^,^^81^^,^^84^, ^85^, ^86^, ^87^^,^^89^, ^90^, ^91^^,^^94^^,^^96^, ^97^, ^98^, ^99^ A similar incidence between intervention and control groups was found in three trials.^65^^,^^74^^,^^100^ Other trials did not register respiratory depression.

## Discussion

From our scoping review of 75 RCTs, we conclude that intrathecally administered morphine effectively reduces postoperative pain for 24 h after spine surgery, thoracic surgery, and orthopaedic surgery of the lower extremity. Moreover, it provides an opioid-sparing effect up until 48 h after surgery. Intrathecal morphine does, however, increase the incidence of pruritus, urinary retention and, to a lesser extent, nausea and vomiting. The efficacy of intrathecal morphine peaks at 8–12 h after surgery and lasts up to 24 h. This is consistent with the pharmacokinetics of intrathecal morphine.^6^

In this review, we included studies comparing intrathecal morphine with different anaesthesia techniques used in daily practice. For spinal and thoracic surgery, we found an overall superior efficacy of intrathecal morphine compared with conventional intrathecal anaesthesia (without opioids) or general anaesthesia in both minor and major surgery. Interestingly, intrathecal morphine was also superior when intrathecal or general anaesthesia was combined with (single shot) long-acting peripheral nerve blocks. In orthopaedic surgery, however, inconclusive results were found. Local infiltration anaesthesia and continuous femoral nerve block were slightly superior to intrathecal morphine with fewer side-effects.

The optimum dose is the lowest dose of any medication that serves the most patients with the fewest side-effects. Although most studies used different doses, some studies were dose finding.^12^^,^^72^^,^^89^ Summarising these studies, the effective dose for spinal surgery would be 0.2–0.3 mg, 0.4–0.5 mg for thoracotomy, 0.3–0.4 mg for minimally invasive thoracic surgery, and 0.1–0.2 mg for orthopaedic surgery. A higher dose can sometimes result in higher efficacy,^7^^,^^12^^,^^13^^,^^81^^,^^89^^,^^90^^,^^103^ but also carries the risk of a higher frequency of side-effects.^7^^,^^89^ If the side-effects are treated pre-emptively, we could probably even use a higher dose to achieve greater efficacy.

Side-effects such as pruritus, PONV and urinary retention may occur.^104^ Urinary retention is not apparent if the patient has a urinary catheter for the first 24 h after the intrathecal morphine injection, which was standard practice in many trials. Urinary retention could be problematic in fast-track orthopaedic surgery. Monitoring of urinary retention is advisable when there is no urinary catheter used after intrathecal morphine. The reported incidence of adverse events was similar to control groups in spinal and thoracic surgery but was increased in lower extremity orthopaedic surgery. A possible dose-dependent increased risk was previously found with a suggested dose threshold of 100 μg.^7^^,^^12^^,^^13^ Local infiltration analgesia is as effective as low-dose intrathecal morphine with fewer side-effects.^13^^,^^71^^,^^76^^,^^80^^,^^91^

A rare but potentially severe adverse effect of intrathecal morphine is delayed respiratory depression. For late respiratory depression, a sufficient dose needs to reach the medulla oblongata by movement of the cerebrospinal fluid. The half-life time of morphine in cerebrospinal fluid is estimated to be 73–140 min. Low to intermediate doses of intrathecal morphine injected at a lumbar level will predominantly have been metabolised before reaching the medulla oblongata.^105^ The short half-life of intrathecal morphine compared with the liquor flow thus makes late respiratory depression unlikely in low to intermediate doses.

Of the sixty included trials with >2300 patients that studied respiratory depression, only two reported a case of early respiratory depression, one case in each trial.^29^^,^^56^ *No cases of late respiratory depression were reported.* In one trial, 8 μg kg^−1^ of intrathecal morphine was administered, and a case of early respiratory depression was registered, but patients with intrathecal morphine had better maintenance of postoperative lung volume.^56^ This relatively high dose (higher than the median effective dose for postoperative analgesia in thoracic surgery) could also explain the occurrence of respiratory depression in the older literature. Doses up to intrathecal morphine 4 mg were often used in the past.^46^ The other trial registered a case of early respiratory depression with only intrathecal morphine 100 μg for endoscopic discectomy.^29^ It was treated with a single dose of naloxone without recurrence. Vascular resorption and transportation from the spinal cord to the respiratory centre could explain this phenomenon, with the temporary high local concentration resulting in respiratory depression. High sensitivity to morphine might have played a role, but this would likely have resulted in recurrent respiratory depression because the effects of intrathecal morphine will outlast those of intravenous naloxone.

The heterogeneity of definitions used for respiratory depression may also account for the high variability in the incidence of respiratory depression reported in the past. A meta-analysis demonstrated recently that the risk for late respiratory depression could be negligible with low doses (<500 μg),^8^^,^^106^ even in older people undergoing hip arthroplasty.^98^^,^^99^ For serious adverse events, intrathecal morphine has a sizeable therapeutic width, which is demonstrated in a recent publication of a 20-fold, pharmacy-generated, intrathecal morphine overdose.^106^

An essential strength of this scoping review is its extensiveness. It does, however, have limitations. The methodological limitations of the included studies constitute a risk of bias because of small numbers per outcome, lack of blinding, and concealment of allocation. Secondly, there is a lot of heterogeneity between the studies for comparisons. These limitations are the reason for using the scoping review format.^26^ Despite these limitations, we provided a solid review of the available literature. It provides a comprehensive overview of available literature on the effectiveness and safety of intrathecal morphine.

## Conclusion

Intrathecal morphine reduced postoperative pain and morphine consumption after spinal surgery, thoracic surgery, and orthopaedic surgery of the lower extremity. The occurrence of pruritus, PONV, and urinary retention does increase with increasing doses of intrathecal morphine. There were no cases of late respiratory depression with low to intermediate doses (<500 μg).

We suggest the administration of intrathecal morphine 100–400 μg in addition to enhanced recovery protocols for spinal and thoracic surgery. Local infiltration anaesthesia has a better risk-to-benefit ratio for orthopaedic surgery, which is most apparent for TKA. If local infiltration anaesthesia cannot be administered, intrathecal morphine may be a good alternative. The risks and benefits of different pain treatment approaches must be considered on a case-by case basis. This scoping review provides some guidance.

## Authors’ contributions

Study design: AT, LG

Design of the work and interpretation of data: AT, LG, SK

Acquisition of data: AT, LG, SK

Analysis of data: AT, LG, RS, SK.

Writing and review of the work: AT, LG, RS, SK.

## Declarations of interest

The authors declare that they have no conflicts of interest.
